# Porcupine Is Not Required for the Production of the Majority of Wnts from Primary Human Astrocytes and CD8+ T Cells

**DOI:** 10.1371/journal.pone.0092159

**Published:** 2014-03-19

**Authors:** Maureen H. Richards, Melanie S. Seaton, Jennilee Wallace, Lena Al-Harthi

**Affiliations:** Department of Immunology/Microbiology, Rush University Medical Center, Chicago, Illinois, United States of America; University of Texas Medical Branch, United States of America

## Abstract

Wnts are small secreted glycoproteins that are highly conserved among species. To date, 19 Wnts have been described, which initiate a signal transduction cascade that is either β-catenin dependent or independent, culminating in the regulation of hundreds of target genes. Extracellular release of Wnts is dependent on lipidation of Wnts by porcupine, a membrane-bound-O-acyltransferase protein in the endoplasmic reticulum. Studies demonstrating the requirement of porcupine for Wnts production are based on cell line and non-human primary cells. We evaluated the requirement for porcupine for Wnts production in human primary astrocytes and CD8+ T cells. Using IWP-2, an inhibitor of porcupine, or siRNA targeting porcupine, we demonstrate that porcupine is not required for the release of Wnt 1, 3, 5b, 6,7a, 10b, and 16a. While IWP had no effect on Wnt 2b release, knockdown of porcupine by siRNA reduced Wnt 2b release by 60%. These data indicate that porcupine-mediated production of Wnts is context dependent and is not required for all Wnts production, suggesting that alternative mechanisms exist for Wnts production.

## Introduction

Wnts are evolutionarily conserved small glycoproteins that initiate the Wnt signaling transduction cascade. The Wnt pathway is involved in many cellular processes including development, proliferation, survival, regeneration, wound healing, and stress responses [1]–[3]. It is initiated by binding of Wnts to frizzled receptors (Fz) initiating either the β-catenin-dependent or β-catenin-independent (calmodulin/Ca2+ and planar polarity) pathway of signal transduction, which culminates in target gene regulation [1], [3]. To date, nineteen Wnts are described in mammals, which are Wnt 1, Wnt 2, Wnt 2b, Wnt 3, Wnt 3a, Wnt 4, Wnt 5a, Wnt 5b, Wnt 6, Wnt 7a, Wnt8a, Wnt 9a, Wnt 9b, Wnt 10a, and Wnt 10b, Wnt 11, and Wnt 16. Wnts are translated and targeted into the endoplasmic reticulum (ER) where they are bound and modified with lipids by the membrane-bound O-acyltransferase Porcupine [4]. Porcupine is an eight transmembrane spanning protein with a carboxy-terminal tail ending within the bilayer of the ER membrane [5]. Porcupine catalyzes the palmitoylation of Wnts which facilitates their secretion to become functionally active. In mice, Wnt3a porcupine allows for lipidation at the cysteine 77 and serine 209 [6], [7]. The lipidated Wnts are then transported to the Golgi where they are bound by the transmembrane protein Wntless. Wntless then shuttles the Wnts to the plasma membrane where they are eventually released into the extracellular space [7]. Findings in mouse and Drosophila, indicate that Wnts require lipidation by porcupine in order to be recognized by WLS [8], [9].

In a seminal paper by Chen et al, small molecule inhibitors of Wnts production were identified using a high stringency cell-based screening strategy in mouse L-cells that stably express the TOPflash construct, a plasmid containing putative TCF/LEF binding sites linked to the firefly luciferase reporter gene. TOPflash activity is an indicator of Wnt/β-catenin activity [10]. Five compounds termed Inhibitors of Wnt Response (IWR) and four compounds termed Inhibitors of Wnt Production (IWP) were identified. Recent published work by the same group has identified an additional 13 IWP compounds [11]. In the original work, they showed that IWP compounds were capable of inhibiting TOPflash activity in response to Wnt1 and Wnt2. IWP-2 was found to block several Wnt-dependent processes including, phosphorylation of LRP5/6, phosphorylation of disheveled2, and accumulation of β-catenin in mouse- L cells. This was not Wnt3a specific as levels of lipidated Wnt5a were also decreased in IWP-2 treated cells. IWP-2 mediates its effect through inhibition of porcupine [10]. If porcupine was overexpressed in IWP-2 treated cells, then lipidation levels were returned to normal levels. L-Wnt3a cells treated with IWP-2 still secreted Wnts but they were not functional as they lacked palmitoylation. In addition, IWP-2 does not alter localization of porcupine to the ER or induce destruction of porcupine indicating that IWP either inhibits the porcupine active site or regulates porcupine [10]. These studies identified IWP-2 as an inhibitor of Wnt production, which is a valuable tool for studies requiring inhibition of Wnts. The consensus is that porcupine is required for Wnt production.

Because many of the studies detailing the complex process of Wnts production are largely based on human cell lines, drosophila, Zebra fish, and mice, we evaluated the requirement for porcupine in Wnts production and efficacy of IWP-2 to inhibit Wnt production and activity in human primary-progenitor-derived astrocytes (PDAs), Human Fetal Astrocytes (HFAs), a human astrocytic cell line (U138), and primary human CD8+ T cells. We demonstrate that IWP-2 does not inhibit Wnt production in these cells and that knockdown for porcupine does not alter the majority of Wnts production, except for Wnt2b. These findings are significant because they indicate that requirement for porcupine for Wnt production is context dependent and also depends on the type of Wnt in question. Most importantly, pharmacological inhibitors of porcupine may not be an effective strategy to block Wnt release in human cells.

## Materials and Methods

### Ethics Statement

Research involving human subjects was conducted in accordance with institutional and U.S. government guidelines on human research. The study was approved by Rush Institutional Review Board (IRB) under protocol number L06080703-CRO1. All donors signed a consent form and a HIPPA form to allow for blood draw. The signed documents were kept in a binder in the lab. The study was performed without identifying the blood donors.

### Culture of Human PDAs, HFAs, U138s, and L-Wnt3a Cells

Human fetal astrocytes (HFA), isolated at approximately 20 weeks of gestation, were purchased from Lonza (Lonza Biologics, Portsmouth, NH). U138MG cells were obtained from ATCC (ATCC HTB-16, Manassas VA) and cultured as described in ([12]). Human Progenitor-Derived Astrocytes (PDA) were generated from neural progenitor cells, as previously described ([13]). Briefly, progenitor cells were provided by Dr. Eugene Major (National Institute of Neurological Disorders and Stroke, National Institutes of Health, Bethesda, MD (NIH) and seeded on poly-D-lysine- coated T-75 tissue culture flasks at 2×10^6^ cells/flask. Cells were maintained in progenitor medium consisting of neurobasal media (Life Technologies Invitrogen, Carlsbad, CA) supplemented with 0.5% bovine albumin (Sigma, St. Louis, MO), neurosurvival factor (Lonza), N2 components (Life Technologies Invitrogen), 25 ng/ml fibroblast growth factor, 20 ng/ml epidermal growth factor (R&D Systems, Minneapolis MN), 50 μg/ml gentamicin (Lonza) and 2 mM L-glutamine (Life Technologies Invitrogen). To induce differentiation, progenitor medium was replaced with PDA medium containing DMEM (Life Technologies Invitrogen) supplemented with 10% heat-inactivated FBS (Sigma), 2 mM L-glutamine, and 50 μg/ml gentamicin. L-Wnt3a (ATCC CRL-2647) were cultured in complete DMEM with 0.4 mg/ml G-418. Media was supplemented every three days.

### Culture and Activation of Human Peripheral Blood Mononuclear Cells (PBMCs) and Isolation of CD8+ T cells

PBMCs were isolated from venous blood of healthy donors using lymphocyte separation medium (Lonza Biologics, Portsmouth, NH) and density centrifugation. PBMCs were then cultured at 1×10^6^ cells mL of RPMI 1640, 10% heat-inactivated fetal bovine serum (FBS), 200 mM L-glutamine, and 1% penicillin/streptomycin (Lonza Biologics, Portsmouth, NH). Cells were activated with 1 μg/mL anti-CD3/anti-CD28 antibodies (BD Biosciences, San Jose, CA) and 100 units of recombinant human IL-2 (AIDS reagent program, Germantown, MD) for three days. In some cases CD8+ T cells were further isolated from PBMCs. CD8+ T cells were isolated using an untouched CD8+ T cell Isolation Kit (Miltenyi Biotec, Germany) on an AutoMACS Separator (Miltenyi Biotec, Germany). CD8+ T cells were cultured for three days with 1 μg/mL anti-CD3/anti-CD28 antibodies (BD Biosciences, San Jose, CA) and 100 units of recombinant human IL-2 (AIDS reagent program) at 1×106 cells/mL of RPMI 1640. In some experiments, cells were stained with carboxyfluoresceinsuccinimidyl ester (CFSE, Invitrogen Life Technologies) prior to culture and treatment with 5 μM IWP-2. Three days later, cells were run on a BD FACSVerse flow cytometer (BD Biosciences) to determine percentage of proliferating cells.

### Transfection with TOPflash and Dual Luciferase Assay

To measure β-catenin-dependent signaling activity, 5×10^6^ PBMCs cells were transfected with 10 μg TOPflash reporter construct (Millipore, Billerica, MA) or FOPflash and renilla plasmid using the Amaxa nucleofection protocol (Amaxa, Gaithersburg, MD), as recommended by the manufacturer. PDA and L-Wnt3a cells were transfected with 3 μg TOPflash reporter construct or FOPflash and renilla plasmid using Lipofectamine (Life Technologies Invitrogen) according to manufacturer’s instructions. Three days later, construct reporter activity was performed using a dual-luciferase reporter assay using 10 to 20 μl of lysate (Promega, Madison, WI). Briefly, the Relative Light Units (RLUs) were normalized to a Renilla luciferase control or μg/ml of protein as indicated. The total protein concentration was measured using a Pierce bicinchoninic acid (BCA) protein assay kit (Thermo Scientific, Waltham, MA).

### siRNA Knockdown of Porcupine

PDAs and U138s were transfected with On-Target plus Smartpool siRNAs (Thermo Scientific) specific for porcupine or scrambled siRNA using Lipofectamine siRNAmax according to the manufacturer’s protocol (Life Technologies Invitrogen). The cells were approximately 60 to 70% confluent at the time of transfection. Efficiency of transfection was confirmed at 48 hours by qRT-PCR and by western blot at 72 and 96 hours.

### Western Blot for Porcupine, Wnt 1, 2b, 3, 5b, 10b and 6, 10a, and 16

Porcupine antibody was purchased from Millipore (Billerica, MA) and all Wnts antibodies were purchased from Abcam (Cambridge, MA). Western blotting was performed according to a standard protocol at the following concentrations of antibodies: Porcupine clone 15G12.1 at 1∶1000, Wnt 1 clone ab15251 at 1∶500, Wnt2b clone ab50575 1:1,000, Wnt3 clone ab32249 at 1∶500, Wnt5b clone ab94914 1:500, Wnt 10b clone ab70816 at 1∶1,000, Wnt6 clone ab50030 at 1∶2000, Wnt7a clone ab100792 at 1∶500, Wnt10a clone ab97469 at 1∶1000, and Wnt16 clone ab64461 at 1∶1000. Secondary antibody conjugated to horseradish peroxidase (HRP) at 1∶50,000 (Cell Signal Technology) was used. The membranes were developed with SuperSignal West Femto maximum-sensitivity substrate (Thermo Sciences).

### Statistics

Comparisons between groups were analyzed using unpaired Student’s t test. p values less that 0.05 were considered significant. All error bars are representative of standard deviation within the sample group. Experiments were repeated at least three times.

## Results

**Figure 1:**
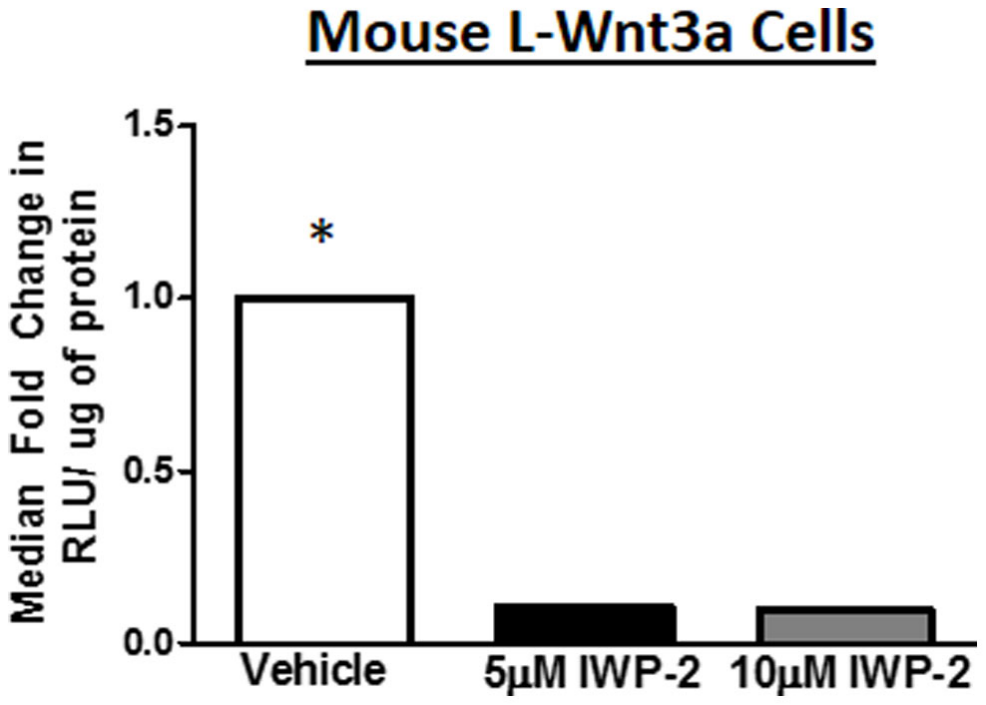
IWP-2 inhibits Wnt mediated β-catenin activity in mouse L-Wnt3a cells. Mouse L-Wnt3a cells were cultured according to ATCC recommendations. Cells were then transfected with TOPflash or vector control and treated with Vehicle (DMSO), 5 μM IWP-2, or 10 μM IWP-2. Three days later a luciferase assay was performed and data was normalized to vector control. Data is representative of three separate experiments. *p≤0.05 in comparison to vehicle.

Previous studies indicated that porcupine is required for Wnt production in drosophila, mouse, and human cells including HEK293, mouse fibroblast L cells, and NIH3T3 cells [1]. To determine whether this requirement is context dependent, we evaluated the efficacy of IWP-2 in inhibiting Wnt production in human primary astrocyte and CD8+ T cells. Firstly, we transfected L-Wnt3a cells with a Wnt/β-catenin pathway responsive firefly luciferase reporter plasmid (TOPflash) prior to treatment with IWP-2 vehicle (10 μM DMSO), 5 μM IWP-2, or 10 μM IWP-2. Consistent with data published by Chen et. al., IWP-2 treatment inhibited Wnt mediated β-catenin activity by 60% in 5 μM IWP-2 and 40% in 10 μM IWP-2 **(** **Fig. 1A** **)** [10]. As 5 μM IWP-2 was better able to reduce β-catenin activity and 5 μM was the dose utilized in previous publications this is the dose we utilized in our studies.

**Figure 2:**
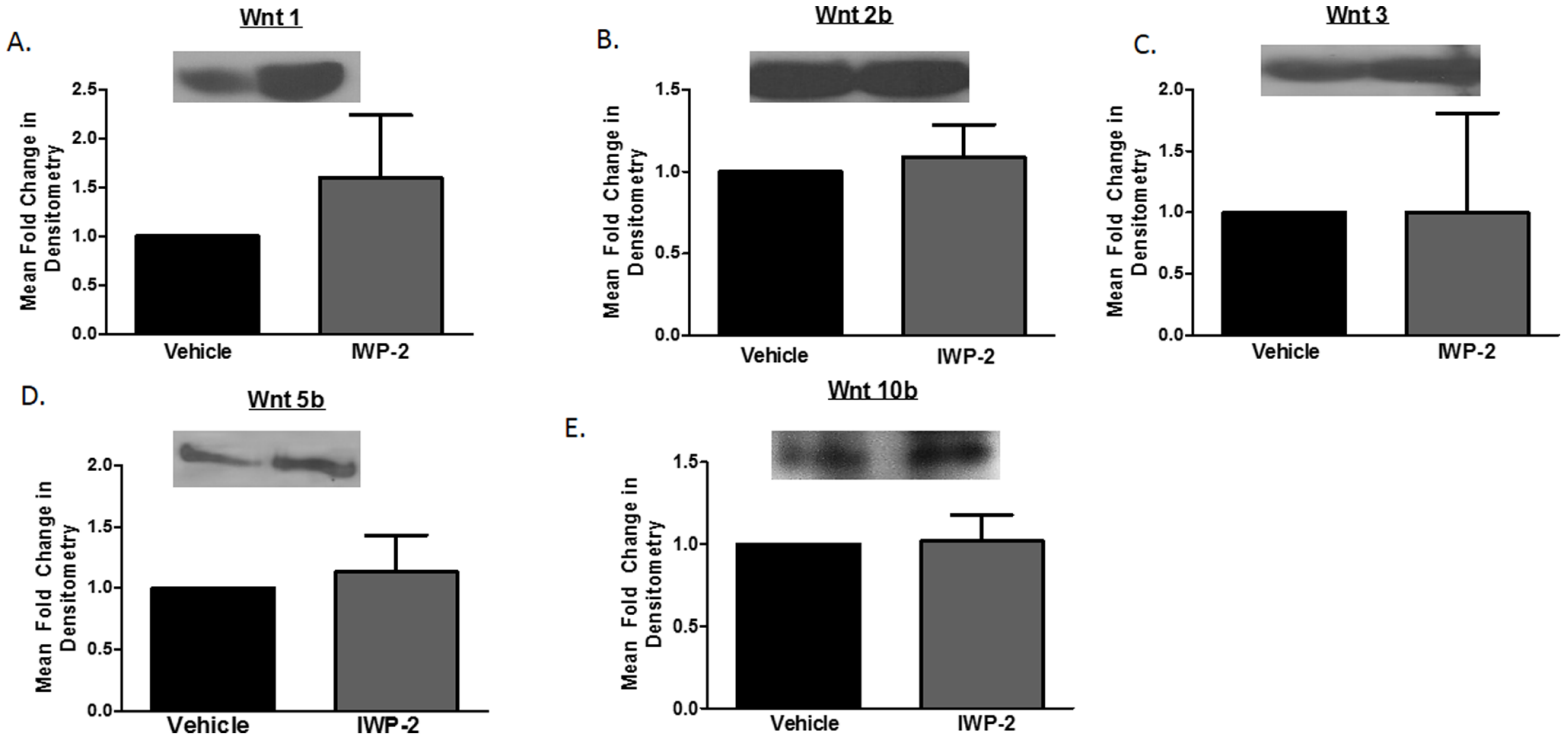
IWP-2 does not inhibit production of Wnts from human astrocytes. PDAs were treated with 5 μM IWP-2 or vehicle (10 μM DMSO). Twenty-four hours later, the media was changed and the cells were treated again with 5 μM IWP-2 for two additional days. On the third day, the supernatant was collected and western blot was performed on 25 μl of supernatant to determine presence of Wnts 1 (A), 2b (B), 3(C), 5b (D), and 10b (E).

Human astrocytes express significant level of endogenous Wnt/β-catenin activity and as such they are an ideal tool to study Wnts production in a system that does not rely on artificial over expression of Wnts [14], [15]. PDAs release Wnt 1, 2b, 3, 5b, and 10b **(** **Fig. 2A–E** **)**. To assess whether IWP-2 can inhibit Wnts production, we treated PDAs with 5 μM of IWP-2 every day for three days. The supernatant was replaced after the first day and cells were re-treated with IWP-2 to allow for accumulation of Wnts. Twenty-four hours after the last IWP-2 treatment, supernatant was collected and a western blot was performed to detect secreted Wnts (**Fig. 2A–E**). Treatment with IWP-2 did not inhibit production of Wnt 1**(** **Fig. 2A** **)**, 2b **(** **Fig. 2B** **)**, 3 **(** **Fig. 2C** **)**, 5b **(** **Fig. 2D** **)**, or 10b **(** **Fig. 2E** **)**. These data indicate that IWP-2 did not alter Wnt levels as evaluated by WB.

**Figure 3:**
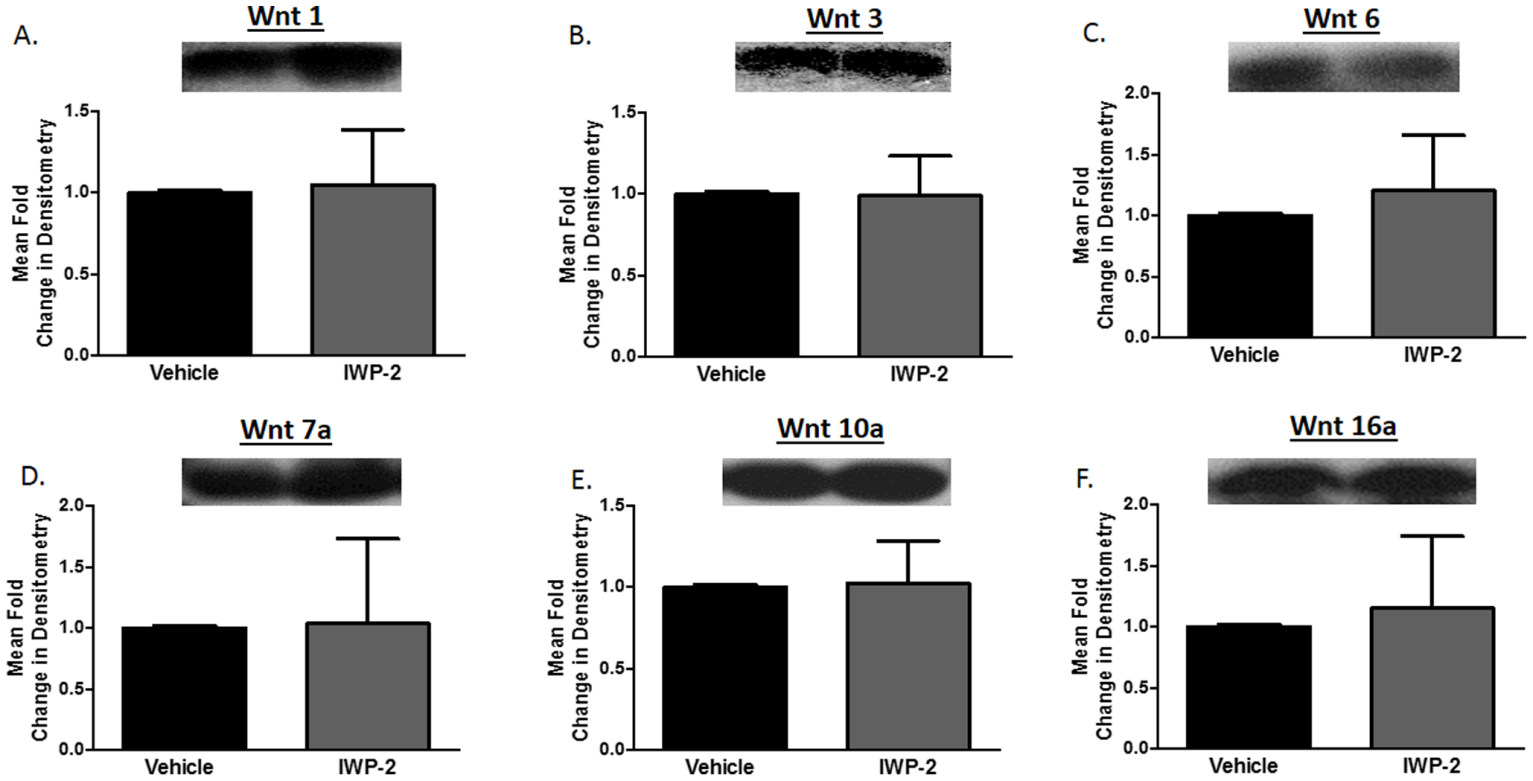
IWP-2 does not inhibit Wnt production from primary human CD8+ T cells. CD8+ T cells were isolated from PBMCs from healthy donors by negative selection and subsequently activated with 1 μg each anti-CD3/anti-CD28 then propagated in presence of 100 units/ml IL-2 for three days. On the third day supernatant was collected and 25 μl was analyzed by western blot for presence of Wnt 1 (A), 3 (B), 6 (C), 7a (D), 10a (E), and 16a (F).

To assess whether the inability of IWP-2 to inhibit Wnt production is astrocyte-specific, we evaluated the impact of IWP-2 on Wnts production in primary human CD8+ T cells. CD8+ T cells secrete Wnts 1, 3, 6, 7a, 10a, and 16 **(** **Fig. 3A–F** **)**. CD8+ T cells were activated by anti-CD3/CD28 co-stimulation and treated with 5 μM IWP-2 for three days. After the third day the supernatant was collected and assessed for Wnt protein. IWP-2 treatment of CD8+ T cells did not alter the expression of any of the Wnts **(** **Fig. 3A–F** **)**. These data demonstrate that IWP-2 treatment does not inhibit production of Wnts in two human primary cell types, astrocytes and CD8+ T cells.

**Figure 4:**
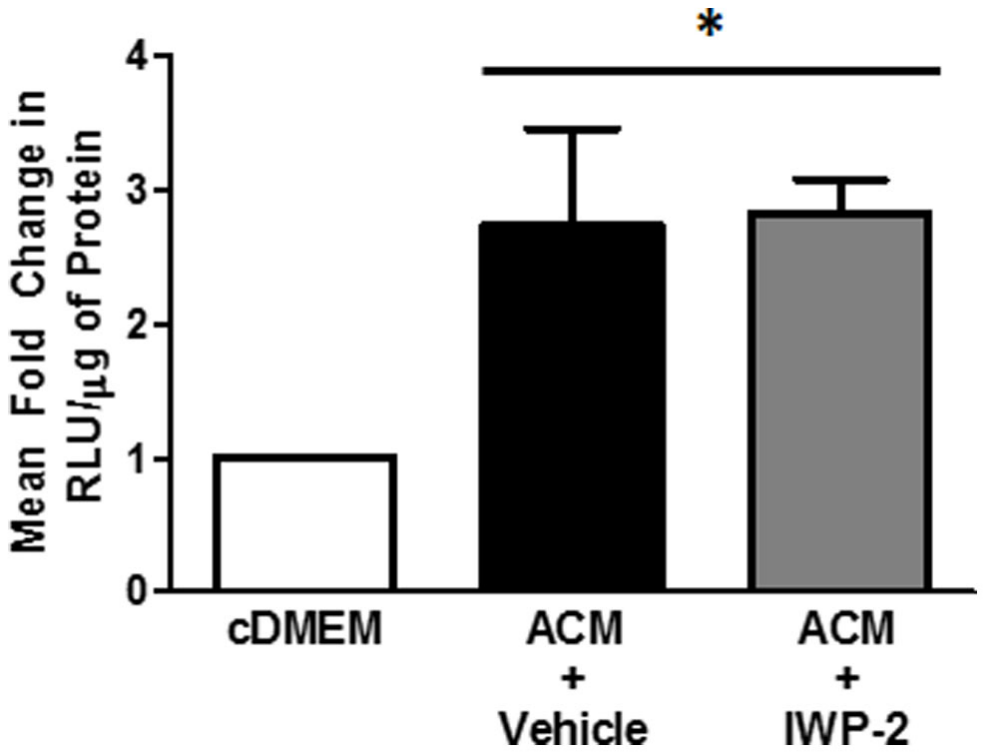
Wnts secreted from HFAs treated with IWP-2 are functional. HFAs were treated with 5 μM IWP-2 or vehicle (10 μM DMSO). Twenty-four hours later, the media was replaced with cDMEM and HFAs were treated with 5 uM IWP-2 each day for 2 days or vehicle. PBMCs were isolated from a healthy donor and activated overnight with 1 μg each anti-CD3/anti-CD28 and 100 units/ml IL-2. PBMCs were then transfected with either TOPflash or FOPflash, a vector control. PBMCs were then cultured with supernatant from IWP-2 or DMSO treated HFAs (ACM). Three days later, β-catenin activity was determined by luciferase assay. Data is representative of three PBMC donors. *p≤0.05.

Chen et al previously showed that IWP-2 inhibited Wnt production and function through inhibition of porcupine [10]. Porcupine is responsible for lipidation of Wnts in the endoplasmic reticulum allowing for Wnts to bind Wntless and be transported from the Golgi to the plasma membrane [7]. Other studies have shown that acylation by porcupine is necessary for Wnt function after production as genetic deletion of porcupine is embryonic lethal [5], [16]–[20]. For example, if the Cys77 loci is not acylated in Wnt3a it is unable to bind frizzled and has reduced signaling activity [21]. To determine whether porcupine is important for Wnts to function in primary human cells, we transfected human PBMCs with TOPflash, treated the cells with supernatant from IWP-2 treated human fetal astrocytes (e.g. astrocyte conditioned media/ACM), and measured TOPflash activity in PBMCs 3 days post-treatment. We found that ACM induced β-catenin signaling in PBMCs by 2.5-fold regardless of whether the ACM was from HFAs treated with IWP-2 or vehicle (DMSO) **(** **Fig. 4** **)**. These data confirm that IWP-2 treatment does not inhibit Wnt ligand secretion or function, as these Wnts can in turn induce β-catenin-mediated signaling as evaluated by TOPflash activity.

**Figure 5:**
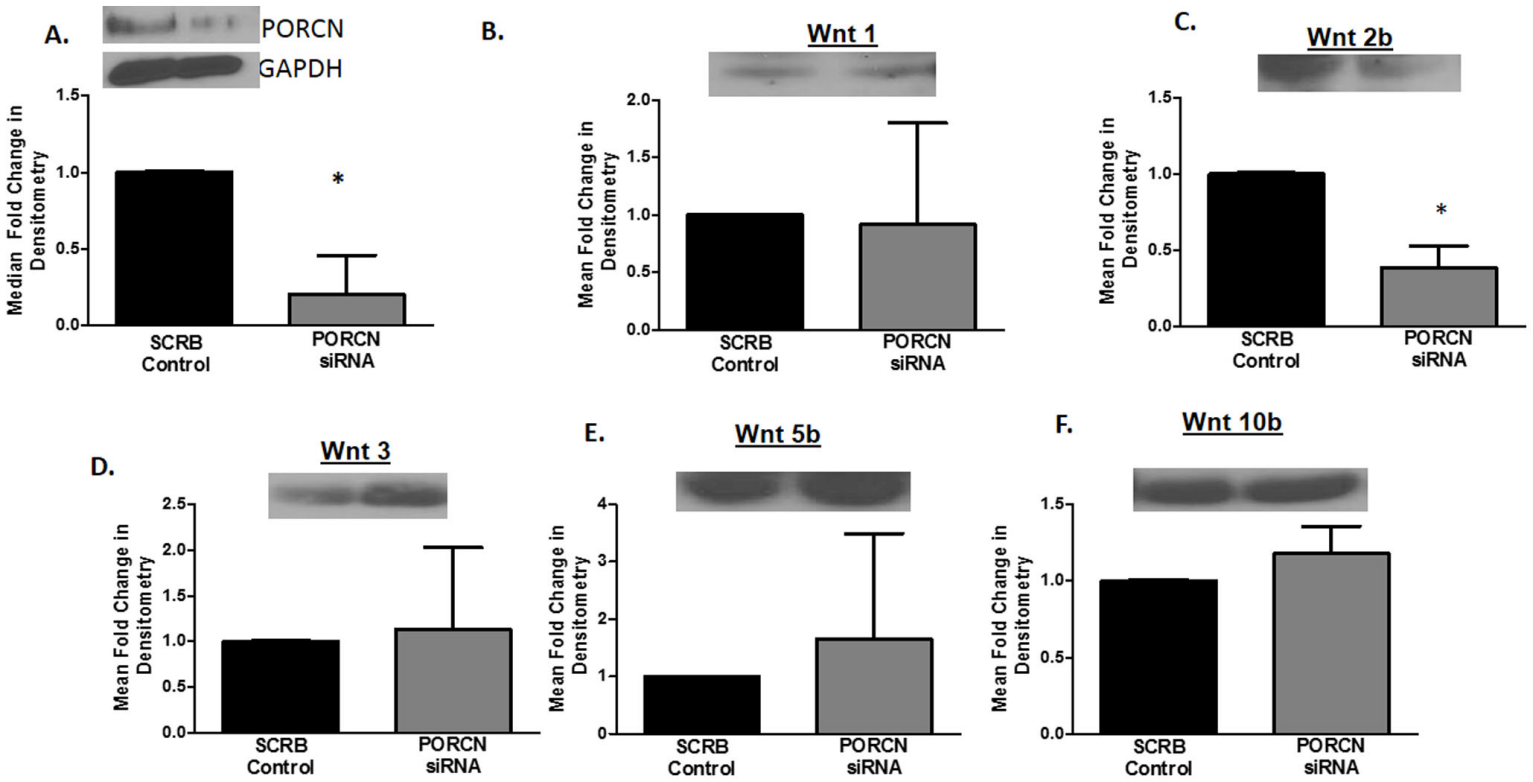
PORCN is not essential for Wnt production by PDAs. PDAs were transfected with smartpool ON TARGET plus siRNA specific for human porcupine or scrambled siRNA. Forty eight hours after transfection cells were lysed and analyzed by western blot to confirm knockdown of porcupine (A). Supernatant was collected from cells at 96 hours post-transfection and analyzed by western blot to determine production of Wnt ligands (B–F). Data is representative of three separate experiments, *p≤0.05.

**Figure 6:**
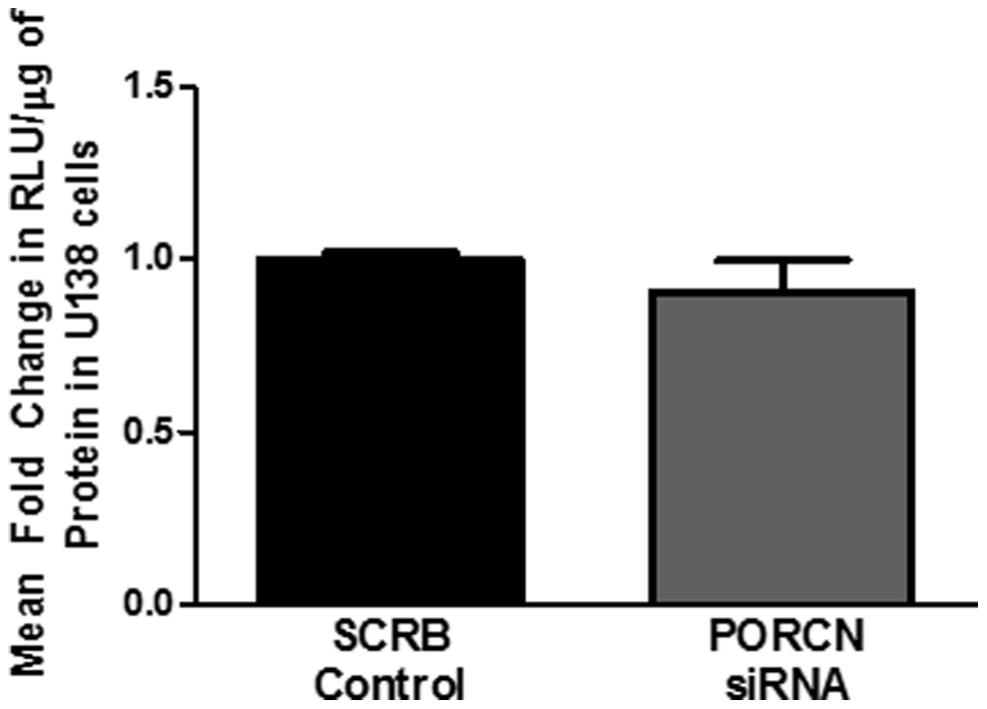
Wnt/β-catenin activity is not altered by porcupine in U138-MG Cells. U138 cells were transfected with either a scrambled siRNA and TOPflash or FOP, and either siRNA specific for porcupine and TOP flash or FOP. 72 hours later β-catenin activity was determined by luciferase assay. This was performed three times.

To better define the role of porcupine in Wnt production in human PDAs, we transfected human PDAs with siRNA- specific for porcupine. Knockdown of porcupine protein was confirmed at 48 hours (**Fig. 5A**) and the supernatant was collected 24 hours later to determine efficacy of Wnts production by Western blot. Although we were able to knockdown greater than 90% of porcupine protein, Wnts 1, 3, 5b, and 10b were not inhibited by this remarkable reduction in porcupine expression **(** **Fig. 5** **B, D–F)**. However, Wnt2b production was inhibited by porcupine knockdown by 60% in comparison to scrambled siRNA **(** **Fig. 5C** **)**. We further confirmed these data in U138s transfected with TOPflash and siRNA for PORCN or scrambled siRNA. Knockdown of porcupine did not alter TOPflash activity in U138s (**Fig. 6**). These data indicate that porcupine is not necessary for production of most Wnt proteins from astrocytes or for β-catenin-mediated transcriptional activity elicited by Wnt ligands. Porcupine is necessary for production of Wnt 2b, but other Wnts are able to compensate for the loss of Wnt 2b thereby not altering β-catenin activity.

## Discussion

We used both the small molecule IWP-2 discovered by Chen et al [10] and siRNA targeting porcupine to assess the impact of porcupine on Wnt ligand secretion in human primary astrocytes (PDAs and HFAs), an astrocytic cell line (U138), and primary human CD8+ T cells. We show through knockdown of porcupine that it is not required for the production of Wnt 1, 3, 5b, and 10b, but it was required for the release of Wnt 2b. Interestingly, IWP-2 molecule was not effective in inhibiting Wnt ligand release in these cells. The pathway of Wnt production and secretion is not fully elucidated. Studies focusing on Wnt secretion have been hampered by the fact that Wnt secretion and activity is necessary for normal development as inhibiting β-catenin activity results in an embryonic lethal phenotype. Additionally, dysregulation of Wnt secretion is linked to a host of disorders including cancer, cardiac pathophysiology, Alzheimer’s disease and others [1], [3], [22]–[25]. Therefore, methods of inhibiting Wnt secretion typically rely on pharmacologic agents (such as small molecule inhibitors) or genetic manipulation of the secretory pathway.

Porcupine has been identified as a key player of Wnt production in several mouse and drosophila studies. However, data regarding porcupine activity is conflicting. When porcupine is inhibited in vivo the outcome is a host of developmental disorders, most notably Goltz Syndrome which causes focal dermal hypoplasia. Conversely, when porcupine is overactive cancerous cell growth occurs [1], [8], [17], [26]. Additionally, while porcupine has been shown to be necessary for the palmitoylation of Wnt 3a in mice it does not appear to be necessary for modifications to the cysteine residue of Wnt1 in mice. Thereby indicating that another enzyme, as yet unknown, may be required for Wnt 1 production [5], [19].

Porcupine may have additional functions beyond modification of Wnts for production. Studies have recently shown that porcupine may “moonlight” in a Wnt independent pathway that regulates proliferation of cancerous cells. Covey et. Al. showed that inducible knockdown of porcupine by several siRNAs lead to delayed growth of established MDA-MB231 cancers in mice [27]. There is increasing evidence that highly conserved proteins may perform multiple different functions utilizing the same protein domain. Porcupine is highly conserved and was initially found in drosophila. While it plays a role in production and activity of some Wnts, our data indicate that it is unnecessary for production and activity of Wnts 1, 3, 5b, and 10b in human astrocytes, and only effective in the production of Wnt 2b. Wnt2b is briefly expressed in the primitive streak during gastrulation and in several organs during development but appears to be redundant as mice lacking Wnt2b are viable, fertile and have a normal life span. In fact, only mice lacking both Wnt2 and Wnt2b display any phenotype [28], [29]. Our data indicate that despite effective knockdown of porcupine and inhibition of Wnt2b in astrocytes, TOPflash activity is not affected. This may be due to the redundant nature of Wnt ligands with Wnts 1, 3, 5b, and 10b promoting β-catenin activity in the absence of Wnt2b. How Wnt2b interacts with porcupine is currently unknown, however our data indicates that porcupine is necessary for Wnt2b production in human astrocytes and CD8+ T cells.

In order to fully elucidate the role of Wnt ligands we must be able to selectively inhibit their activity and production. A better understanding of the Wnt production pathway for each of the 19 known human Wnts is necessary before global inhibitors can be used. Our data and data from others indicate that different species may have developed redundant pathways for Wnt production that may vary from cell type to cell type. Further investigation of the Wnt secretory pathway in primary cells is necessary before the full impact of Wnts can be appreciated.
